# Probiotics' effects on the incidence of nosocomial pneumonia in critically ill patients: a systematic review and meta-analysis

**DOI:** 10.1186/cc11398

**Published:** 2012-06-25

**Authors:** Kai-xiong Liu, Ying-gang Zhu, Jing Zhang, Li-li Tao, Jae-Woo Lee, Xiao-dan Wang, Jie-ming Qu

**Affiliations:** 1 Department of Pulmonary Medicine, Huadong Hospital, Shanghai Medical School of Fudan University, 221 Yananxi Road, Shanghai 200040, China; 2 Department of Pulmonary Medicine, Zhongshan Hospital, Shanghai Medical School of Fudan University, 180 Fenglin Road, Shanghai 200032, China; 3 Department of Anesthesia & Preoperative Care, University of California, San Francisco, 505 Parnassus Ave, San Francisco 94143, USA

## Abstract

**Introduction:**

To evaluate the efficacy of probiotics in preventing nosocomial pneumonia in critically ill patients.

**Methods:**

We searched PubMed, EMBASE, and the Web of Science for relevant studies. Two reviewers extracted data and reviewed the quality of the studies independently. The primary outcome was the incidence of nosocomial pneumonia. Study-level data were pooled using a random-effects model when *I^2 ^*was > 50% or a fixed-effects model when *I^2 ^*was < 50%.

**Results:**

Twelve randomized controlled studies with a total of 1,546 patients were considered. Pooled analysis showed a statistically significant reduction in nosocomial pneumonia rates due to probiotics (odd ratio [OR]= 0.75, 95% CI 0.57 to 0.97, *P *= 0.03, *I^2 ^*= 46%). However, no statistically significant difference was found between groups regarding in-hospital mortality (OR = 0.93, 95% CI 0.50 to 1.74, *P *= 0.82, *I^2 ^*= 51%), intensive care unit mortality (OR = 0.84, 95% CI 0.55 to 1.29, *P *= 0.43, *I^2 ^*= 0%), duration of stay in the hospital (mean difference [MD] in days = -0.13, 95% CI -0.93 to 0.67, *P *= 0.75, *I^2 ^*= 46%), or duration of stay in the intensive care units (MD = -0.72, 95% CI -1.73 to 0.29, *P *= 0.16, *I^2 ^*= 68%).

**Conclusions:**

The use of probiotics was associated with a statistically significant reduction in the incidence of nosocomial pneumonia in critically ill patients. However, large, well-designed, randomized, multi-center trials are needed to confirm any effects of probiotics clinical endpoints such as mortality and length of ICU and hospital stay.

## Introduction

Nosocomial pneumonia (NP) is a common complication in critically ill patients, particularly in patients who are intubated for more than 48 hours, and NP is responsible for significant in-hospital morbidity and mortality [1-3]. When mechanically ventilated patients develop NP, it is known as ventilator-associated pneumonia (VAP) [1,2]. Multiple hospital-associated risk factors for NP have been identified. These risk factors are thought to contribute to increased bacterial colonization of the aerodigestive tract and facilitate the entry of pathogenic bacteria into the lower respiratory tract [4].

Considerable efforts have been made to evaluate methods for reducing NP. For example, selective digestive tract decontamination in critically ill patients has been shown to reduce the occurrence of NP; however such decontamination has also been associated with increased rates of antimicrobial resistance [5,6]. Several experimental and clinical studies have suggested a promising effect of probiotics on preventing NP in critically ill patients [7-10].

Probiotics are commercially available microorganisms that when ingested as individual strains or in combination may offer potential health benefits to the host [11]. Prebiotics are non-digestible sugars that selectively stimulate the growth of certain bacteria colonies. The combination of pre- and probiotics has been designated as synbiotics. It is hypothesized that probiotics could potentially reduce the incidence of NP in critically ill patients through various local and systemic effects that minimize colonization by more virulent species or optimize host immune defenses. These effects include reducing overgrowth of potentially pathogenic microorganisms, enhancing gut barrier function, reducing bacterial translocation, and up-regulation of immune functions [12-19].

To date, clinical research concerning the effects of probiotics in critically ill patients have provided conflicting results, with some suggesting clinical benefit [20-29], and others showing no benefit [30-33]. More recently, one study showed that probiotics therapy led to a significant reduction in VAP rates among treated patients [22]. However, another study showed that daily prophylactic administration of probiotics was not effective for critically ill patients, notably for those with non-severe sepsis [30].

Therefore, we performed a systematic literature review and meta-analysis to investigate the effects of probiotics in critically ill patients using incidence of NP as the primary outcome, and mortality, length of stay in the ICU and in hospital, and adverse outcomes as secondary outcomes.

## Materials and methods

### Data sources and search strategy

To identify studies for inclusion in this review, two authors independently searched PubMed, the Cochrane Central Database of Controlled Trials, and EMBASE for relevant studies published up to January 2012. The search was limited to studies conducted in humans. No language restriction was imposed. Search terms were individualized for each database. Search terms used included: ['pneumonia' OR 'critically ill' OR 'intensive care' OR 'trauma' OR 'pancreatitis' OR 'surgical patients'] AND ['probiotics' OR 'prebiotics' OR 'synbiotics' OR '*lactobacillus*' OR '*bifidobacterium*']. We also searched the proceedings of major relevant conferences, trial databases, the reference lists of identified trials, and major reviews.

### Study selection

Two reviewers (KXL and YGZ) independently screened studies for inclusion, retrieved potentially relevant studies, and determined study eligibility. Any discrepancies were resolved by consensus. Analysis was restricted to double-blind, randomized controlled trials (RCTs). For this meta-analysis, we considered those RCTs that compared administration of probiotics vs. placebo in critically ill patients (such as those admitted to an ICU or having recently undergone abdominal or another major surgical procedure), and that reported the incidence of NP or VAP. Probiotics could be administered either alone or in combination with prebiotics.

### Data extraction

Two authors independently extracted data from all of the enrolled studies. Extracted data included study design (for example, year conducted, sample size), patient characteristics, study methodology (for example, eligibility criteria, method of randomization and blinding), intervention (for example, type of probiotic agent, dose, route of its administration and duration), and clinical outcomes. The primary outcome was the incidence of NP. We used the authors' definitions for NP if they included clinical and radiological criteria. Secondary outcomes were mortality, length of stay in ICU and in hospital, and reports of adverse outcomes.

### Quality assessment

We formally assessed the methodological quality of each trial using the Jadad score [34], which incorporates randomization, blinding, and attrition to derive a score of 0 to 5; higher scores indicate higher quality. Two reviewers (KXL and YGZ) independently appraised the quality of the included trials. Studies were considered to be of low quality if the Jadad score was ≤ 2 and high quality if the score was ≥ 3.

### Statistical analysis

The meta-analysis was performed using Review Manager 5.0 (Cochrane Collaboration, Oxford, UK). We computed pooled odds ratios (ORs) and 95% confidence intervals (CIs) from the adjusted ORs and 95% CIs reported in the observational studies. We used Cochrane Q and *I^2 ^*statistics to assess the heterogeneity of study results. We predefined heterogeneity as low, moderate or high with *I^2 ^*values above 25%, 50%, and 75%, respectively. In the analysis of heterogeneity, we considered a *P-*value < 0.10 statistically significant. Study-level data were pooled using a random-effects model when *I^2 ^*was > 50% or a fixed-effects model when *I^2 ^*was < 50%. Publication bias was assessed by a funnel plot using the occurrence of NP as an endpoint.

## Results

**Figure 1:**
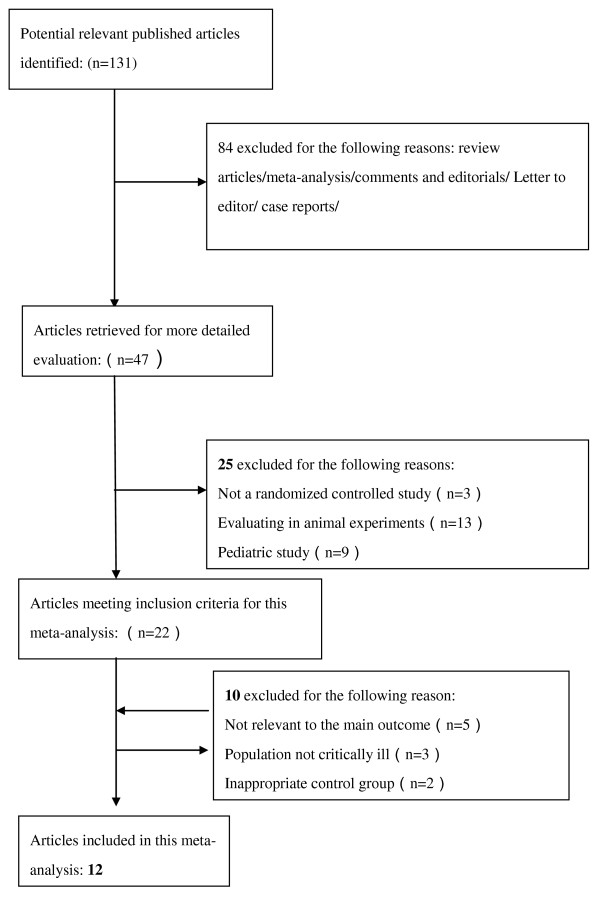
**Flow chart of study selection**. Pooled ORs were calculated using the Mantel-Haenszel (M-H)Estimator. Study-level data were pooled using a random-effects model when *I^2 ^*was > 50% or a fixed-effects model when *I^2 ^*was < 50%.

Our search retrieved a total of 131 references. After applying the inclusion criteria, twelve studies were included in this meta-analysis [22-33]. A flowchart for the studies evaluated and the reasons for exclusion are shown in Figure 1.

### Study characteristics

Characteristics of the included studies are summarized in Table 1. A total of 1,546 critically ill patients were included in these studies. All studies were published from 2002 to 2011. Trials were conducted in a diverse array of countries. Most of the trials were carried out at a single center. Four studies recruited patients in general ICUs [22,30-32], eight studies recruited patients in surgical ICUs [23-29,33], one study recruited patients who were scheduled for liver transplantation [26], and one study recruited patients with severe acute pancreatitis [33]. Seven of the twelve clinical trials enrolled patients who required mechanical ventilation (MV) from medical and surgical ICUs [22,23,28-32]. The frequency of probiotic administration ranged from once to twice a day. In the majority of eligible trials, probiotics were administered via nasogastric or orogastric tube [22,23,28-32] until the patient's discharge from the ICU or death. In some surgical patients, probiotics were administered via nasojejunal tubes [24-27,33]. The average Jadad score of these studies was 3.5 (range 2.0 to 5.0) (Table 2). Results of the meta-analyses that explored the effects of probiotics on clinical outcomes are shown in Table 3.

**Table 1:** Characteristics of the study population in various studies

Study, year	Study design	Population	Disease severity Score	Regimen used	Route of administration/duration of intake
Barraud *et al*., 2010 [30]	SC DBRCT	General/all intubated adult patients under MV ≥ 48 hours	SAPS II:58.6 ± 17.3 vs. 60.5 ± 19.6	Ergyphilus (*Lactobacillus rhamnosus GG, Lactobacillus casei, Lactobacillus acidophilus, Bifidobacterium bifidum*)	Enteral feeding tube/entire period of MV and additional days
Besselink *et al*., 2008 [33]	MC DBRCT	Patients with predicted severe acute pancreatitis	APACHE II: 8.6 ± 4.4 vs. 8.4 ± 4.5	Ecologic 641 (*Lactobacillus acidophilus, Lactobacillus casei, Lactobacillus salivarius,Lactococcus lactis, Bifi dobacterium bifidum*, and *Bifidobacterium* *lactis*)	Nasojejunal tube/28 days
Forestier *et al*., 2008 [31]	SC RCT	General/patients (>18 yrs) requiring MV > 48 hours	SAPS II: 4 5± 16 vs. 44 ± 15	*Lactobacillus caseirhamnosus*	Nasogastric or orogastric tube/until ICU discharge or death
Giamarellos-Bourboulis *et al*., 2009 [23]	MC DBRCT	Surgical/severe multiple organ injuries necessitating emergency tracheal intubation and ventilation support	APACHE II: 19.36 vs. 19.36	Synbiotic2000 FORTE (*Pediococcus pentosaceus, Leuconostoc mesenteroides*, *Lactobacillus paracasei subsp paracasei and Lactobacillus plantarum*)	Nasogastric tube or through gastostomy/15 consecutive days post admission
Kanazawa *et al*., 2005 [24]	SC RCT	Surgical/patients with biliary cancer, scheduled to undergo combined liver and extrahepatic bile duct resection with hepaticojejunostomy	NA	Yakult BL Seichōyaku (*Bifidobacterium breve*Strain Yakult, *Lactobacillus casei *strain Shirota)	Intraoperative jejunal feeding catheter/14 days post-surgery
Knight *et al*. 2009, [32]	SC DBRCT	General/patients (> 16yrs) requiring MV > 48 hours	APACHE II: 17 (12-23) vs.17 (12-22)	Synbiotic2000 FORTE (*Pediococcus pentosaceus, Leuconostoc mesenteroides*, *Lactobacillus paracasei subsp paracasei and Lactobacillus plantarum*)	Nasogastric or orogastric tube/until 28 days after ICU admission, discharge or death
Morrow *et al*., 2010 [22]	SC DBRCT	General/patients (> 19 yrs) requiring MV with an endotracheal tube for at least 72 hours	APACHE II: 22.7 ± 7.5 (8-38) vs. 23.7 ± 8.0 (8-41)	*Lactobacillus rhamnosus GG*	Nasogastric tube/until extubation, tracheostomy placement, or death
Rayes *et al*., 2002 [25]	RCT	Surgical/patients who were scheduled for major abdominal surgery	NA	*Lactobacillus plantarum *299 and fibres	Nasojejunal tube/7 days post-surgery
Rayes *et al*., 2005 [26]	DBRCT	Surgical/patients scheduled for liver transplantation	NA	Synbiotic2000 FORTE (*Pediococcus pentosaceus, Leuconostoc mesenteroides*, *Lactobacillus paracasei subsp paracasei and Lactobacillus plantarum*)	Nasojejunal tube/14 days post-surgery
Rayes *et al*., 2007 [27]	MC DBRCT	Surgical/patients who were scheduled for pancreaticoduodenectomy	NA	Synbiotic2000 FORTE (*Pediococcus pentosaceus, Leuconostoc mesenteroides*, *Lactobacillus paracasei subsp paracasei and Lactobacillus plantarum*)	Oral (pre-surgery) and nasojejunal tube (post-surgery)/1 day pre- to 8 days post-surgery
Spindler- Vesel *et al*., 2009 [28]	SC RCT	Surgical/multiple injury patients requiring MV and at least 4 days stay in ICU	APACHE II: 14 (12-19) vs. NA	Synbiotic2000 FORTE (*Pediococcus pentosaceus, Leuconostoc mesenteroides*, *Lactobacillus paracasei subsp paracasei and Lactobacillus plantarum*)	Intragastric tube/until ICU discharge or death
Tan *et al*., 2011[29]	SC RCT	Surgical/patients with closed head injury only; admission within 24 hours after trauma (18 to 60 yrs)	APACHE II: 14.8 ± 3.6 vs 14.8 ± 3.6	Golden Bifid (*Bifidobacterium* *longum, Lactobacillus bulgaricus*, *and Streptococcus thermophilus*)	Nasogastric tube/21 consecutive days

APACHE: Acute Physiology and Chronic Health Evaluation; DB: double-blind; ICU: intensive care unit; MC: multicenter; MV: mechanical ventilation; NA: not available; RCT: randomized control trial; SAPS: Simplified Acute Physiology Score; SC: single-center.

**Table 2:** Quality of the twelve studies as assessed by the Jadad score [34]

Study	Randomization	Blinding	Withdrawals and dropouts	Quality Score
Barraud *et al*. [30]	2	2	1	5
Besselink *et al*. [33]	2	2	1	5
Forestier *et al*. [31]	2	2	1	5
Giamarellos-Bourboulis *et al*. [23]	1	2	0	3
Kanazawa *et al*. [24]	1	1	0	2
Knight *et al*. [32]	2	2	1	5
Morrow *et al*. [22]	1	2	1	4
Rayes *et al*. [25]	2	0	1	3
Rayes *et al*. [26]	2	1	1	4
Spindler-Vesel *et al*. [28]	1	1	0	2
Tan *et al*.[29]	2	1	1	4

Each article was scored using a five-point scale that evaluates randomisation, blinding and completeness of patient follow-up (Jadad scale). One point was given if the study was described as randomised. An additional point was given if the randomisation method was described and was appropriate (for example, computer-generated table of random numbers), whereas a point was subtracted if the randomisation method was described and inappropriate. Similarly, one point was assigned to studies described as double-blinded, two points were assigned to studies for which the double-blinding method was described and appropriate (for example, identical placebo, active placebo,double-dummy) and zero points were assigned to studies for which the double-blinding method was described and inappropriate. One point was given if the article specified the numbers of and reasons for withdrawals and dropouts.

**Table 3:** Outcome data of the randomized controlled trials included in the meta-analysis (comparison of probiotics versus control)

Study	Incidence of NP/VAP, n/N	ICU mortality, n/N	In-hospital mortality, n/N	Length of ICU stay, median days (range)	Length of hospital stay, median days (range)
Barraud *et al*. [30]	23/87 vs. 15/80 (VAP)	21/87 vs. 21/80	NA	18.7 ± 12.4 vs.20.2 ± 20.8	26.6 ± 22.3 vs. 28.9 ± 26.4
Besselink *et al*. [33]	24/152 vs. 16/144	NA	24/152 vs. 9/144	6.6 ± 17.1vs. 3.0 ± 9.3	28.9 ± 41.5 vs. 23.5 ± 25.9
Forestier et al [31]	24/102 vs. 24/106 (VAP)	NA	NA	NA	NA
Giamarellos-Bourboulis *et al*. [23]	15/36 vs. 16/36 (VAP)	NA	5/36 vs. 10/36	NA	NA
Kanazawa *et al*. [24]	0/21 vs. 1/23	NA	0/21 vs. 0/23	1.3 ± 0.9 vs. 1.3 ± 0.7	36.9 ± 16.4 vs. 47.0 ± 19.2
Knight *et al*. [32]	12/130 vs.17/129 (VAP)	28/130 vs. 34/129	35/130 vs. 42/129	6 (3-11) vs. 7 (3-14)	19 (3-36) vs. 18 (7-32)
Morrow *et al*. [22]	17/68vs. 33/70 (VAP)	NA	12/68 vs.15/70	14.8 ± 11.8 vs. 14.6 ± 11.6	21.4 ± 14.9 vs. 21.7 ± 17.4
Rayes *et al*. [25]	2/30 vs. 6/30	NA	0/30 vs. 0/30	NA	14 ± 4 vs. 16 ± 5.5
Rayes *et al*. [26]	0/33 vs. 1/33	NA	0/33 vs.0/33	8.80 ± 0.9 vs. 10.2 ± 1.8	27.8 ± 2.4 vs. 27.9 ± 2.1
Rayes *et al*. [27]	0/40 vs. 4/40	NA	1/40 vs. 1/40	2 ± 3 vs. 6 ± 12	17 ± 8 vs. 22 ± 16
Spindler-Vesel *et al*. [28]	4/26 vs. 34/87 (VAP)	2/26 vs. 5/87	NA	NA	NA
Tan *et al*.[29]	9/22 vs. 14/21	NA	0/22 vs. 1/21	7.1 ± 3.3 vs.11.3 ± 7.9	NA

ICU: intensive care unit; NA: not available; NP: nosocomial pneumonia; VAP: ventilator-associated pneumonia.

### Nosocomial pneumonia and subgroup analyses

**Figure 2:**
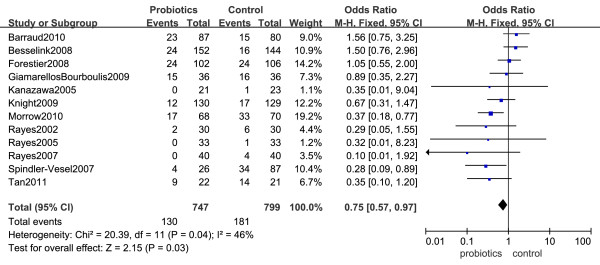
**Forest plot showing the effect of probiotics on the occurrence of nosocomial pneumonia (NP) in critical ill patients**. Pooled ORs were calculated using the Mantel-Haenszel (M-H)Estimator. Study-level data were pooled using a random-effects model when *I^2 ^*was > 50% or a fixed-effects model when *I^2 ^*was < 50%.

Results from twelve trials (1,546 patients) were available to examine the effects of oral probiotics on the incidence of NP. A low level of heterogeneity was found among the identified comparisons (*I^2 ^*= 46%, *P *= 0.04). Pooled analysis showed that the use of probiotics was associated with a statistically significant reduction in the incidence of NP in critically ill patients (OR = 0.75, 95% CI 0.57 to 0.97, *P *= 0.03) (Figure 2).

We also performed subgroup analyses after stratifying trials by critically ill patients requiring MV (see Additional File 1) and surgical critically ill patients (see Additional File 2). Seven studies reported the incidence of VAP [22,23,28-32]. No statistically significant difference in the incidence of VAP was found between patients who received probiotics and patient controls (OR = 0.68, 95% CI 0.42 to 1.11, *P *= 0.12, *I^2 ^*= 54%). We noted a marginally significant beneficial effect of probiotics on reducing the rate of NP in critically ill surgical patients in a meta-analysis that included eight studies (OR = 0.67, 95% CI 0.45 to 1.01, *P *= 0.05, *I^2 ^*= 41%) [23-29,33].

### Overall mortality

**Figure 3:**
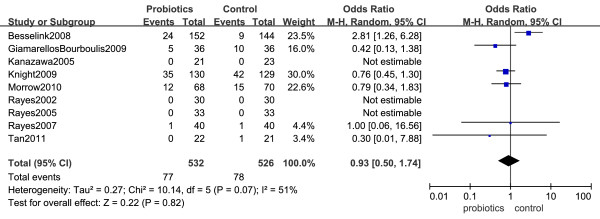
**Forest plot showing the effect of probiotics on in-hospital mortality**. Pooled ORs were calculated using the Mantel-Haenszel (M-H)Estimator. Study-level data were pooled using a random-effects model when *I^2 ^*was > 50% or a fixed-effects model when *I^2 ^*was < 50%.

**Figure 4:**
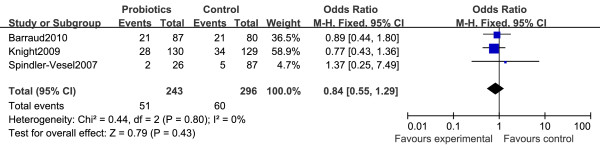
**Forest plot showing the effect of probiotics on ICU mortality**. Pooled ORs were calculated using the Mantel-Haenszel (M-H)Estimator. Study-level data were pooled using a random-effects model when *I^2 ^*was > 50% or a fixed-effects model when *I^2 ^*was < 50%.

Results of nine trials were available for the analysis of mortality during the entire hospital stay [22-27,29,32,33]. A meta-analysis of these trials found that probiotics administration had no effect on overall mortality during the hospital stay (OR = 0.93, 95% CI 0.50 to 1.74, *P *= 0.82) (Figure 3). We did find evidence of statistical heterogeneity for in-hospital mortality (*I^2 ^*= 51%, *P *= 0.07). Only three of the twelve selected RCTs provided information regarding mortality during an ICU stay [28,30,32]. There was no significant difference in ICU mortality between a probiotics group and a placebo group (OR = 0.84, 95% CI 0.55 to 1.29, *P *= 0.43) (Figure 4). There was no heterogeneity between trials (*I^2 ^*= 0%).

### Duration of stay in the hospital

**Figure 5:**
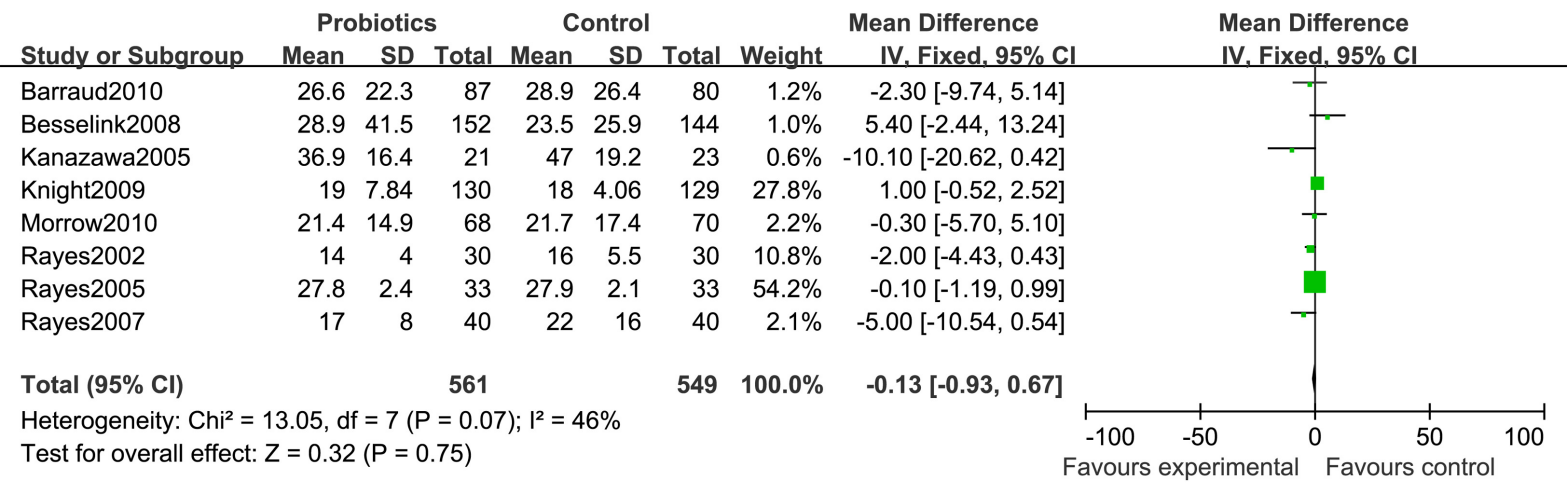
**Forest plot showing the effect of probiotics on length of hospital stay (in days)**. Mean differences were estimated by the inverse variance (IV) approach.

Eight studies were included in the analysis of the length of stay in hospital [22,24-27,30,32,33]. There was no apparent effect of probiotics therapy on the duration of stay in hospital, with a mean difference (MD) of -0.13 days (95% CI -0.93 to 0.67, *P *= 0.75) (Figure 5). A low level of heterogeneity was found among these comparisons (*I^2 ^*= 46%, *P *= 0.07).

### Duration of stay in the intensive care unit

**Figure 6:**
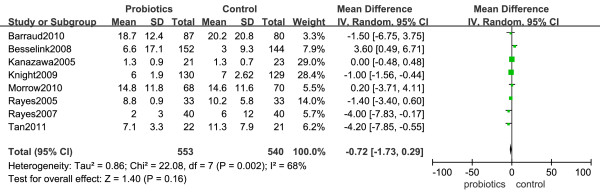
**Forest plot showing the effect of probiotics on length of ICU stay (in days)**. Mean differences were estimated by the inverse variance (IV) approach.

Data from eight studies were included in the analysis of the duration of stay in the intensive care unit [22,24,26,27,29,30,32,33]. There was significant heterogeneity in length of ICU stays (*I^2 ^*= 68%, *P *= 0.002) (Figure 6). There was no significant difference between the compared groups regarding this outcome (MD in days = -0.72, 95% CI -1.73 to 0.29, *P *= 0.16).

### Adverse events

The following adverse events were reported: diarrhea, abdominal cramps and bowel ischemia [22,25-27,32,33]. Data regarding the incidence of diarrhea were provided in six of the twelve included RCTs [22,25-27,32,33]. There was no difference between probiotics and placebo groups in the incidence of diarrhea (OR= 0.85, 95% CI 0.58 to 1.26, *P *= 0.43, *I^2 ^*= 0%). There was no significant difference between probiotics and placebo groups in the incidence of abdominal cramps in the meta-analysis that included only three RCTs (OR = 0.74, 95% CI 0.47 to 1.17, *P *= 0.19, *I^2 ^*= 0%) [25,26,33].

### Publication bias

**Figure 7:**
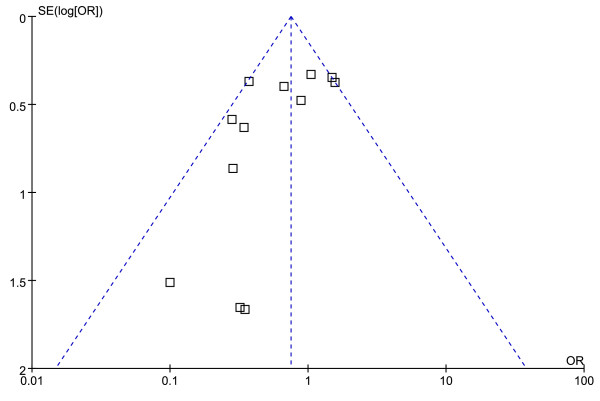
**Funnel plot showing possibility of a small publication bias**. SE, standard error: OR, odds ratio.

Upon visual inspection of the funnel plot for the primary outcome, we found evidence of publication bias (absence of small studies, shown in the right lower corner of Figure 7).

## Discussion

Our meta-analysis found that probiotics administration was associated with statistically significant reduction in the incidence of NP in critically ill patients. However, the pooled results showed that probiotics did not affect overall mortality, or length of stay in the hospital and the ICU, which were the secondary endpoints of the study.

The current meta-analysis is different from previous reviews in several aspects. Although three recent reviews addressed somewhat similar questions, our meta-analysis identified and included more eligible studies than the previous reviews [35-37]. These previous reviews on probiotics administration included studies that recruited patients requiring MV only [35] or studies of surgical patients only [36]. Thus, those meta-analyses were limited to selected populations. Trials of any type of critically ill patients were eligible for our study, and therefore our results are applicable across a wide range of clinical situations that are encountered with critically ill patients. In order to diminish the number of confounding factors, we excluded two studies using chlorhexidine and antibiotic decontamination as control groups, because the use of chlorhexidine in oral care procedures and antibiotic decontamination of the digestive tract were considered to be effective in preventing NP [6,21]. The review by Siempos *et al*. used the data from one trial on the rate of respiratory tract infection as the rate of VAP [35], which may have contributed to an overestimation of the VAP rate and a greater observed treatment effect. This trial has been recently published in a separate study [23] and confirms that there are fewer patients with VAP than with respiratory tract infections. Accordingly, we included the latter study in our meta-analysis. Our results appear similar to the previous reviews by Siempos *et al*. [35] and Pitsouni *et al*. [36], but inconsistent with the results of the systematic review by Watkinson *et al*. [37]. Siempos *et al*. found that administration of probiotics was beneficial in the incidence of both VAP and NP, length of stay in the ICU and colonization rates of *Pseudomonas aeruginosa *in the respiratory tract [35]. Similarly, the meta-analysis by Pitsouni *et al*. demonstrated that probiotics significantly reduced the occurrence of postoperative pneumonia and any infectious complications, as well as the duration of postoperative hospital stay [36]. While we restricted our subgroup analysis to patients in surgical populations, a pooled analysis showed a marginally non-significant reduction in NP in favor of probiotics. In contrast, Watkinson *et al*. pooled eight trials and found that pre- pro- or synbiotics were not associated with any significant change in the outcomes studied, that is, length of ICU stay, hospital mortality and the incidence of nosocomial infection and more specifically incidence of pneumonia [37]. Although there was no statistically significant effect on the incidence of NP in all subgroups, the risk reduction associated with probiotics use was substantial. The reasons for these inconsistent results may partly be due to differences in focus on clinical outcomes.

The results of this meta-analysis should be interpreted carefully based on other considerations. As the diagnosis of pneumonia is a more subjective outcome than mortality or length of stay in the ICU, it may be more subject to bias, and this may in part explain the marked reduction in pneumonia found in these studies. In addition, the definitions of pneumonia varied among different studies, which will affect the true nature of clinical outcomes. In addition, the absence of an effect on secondary outcomes may be from the small number of pooled RCTs and total patients. And lastly, the treatment durations in some studies were likely too short to demonstrate maximal benefits. Consequently, a lack of standard protocols and insufficient numbers of patients may make it difficult to derive conclusive results based on the current meta-analysis.

The potential harm due to probiotics therapy also warrants comment. The numbers of patients with diarrhea or abdominal cramps did not differ between those patients who received probiotics and those who did not in our current meta-analysis. However, a particular concern in critically ill patients is whether their exposure to probiotics places them at risk for developing an invasive infection. There were no reports of bacteremia or sepsis due to probiotics in the studies included in our meta-analysis. In addition, Besselink *et al*. found an increased rate of bowel ischemia and mortality in those patients treated with probiotics [33]. However, a meta-analysis of four RCTs that included severe acute pancreatitis, including the study by Besselink *et al*. [38] demonstrated that probiotics did not significantly influence mortality either favorably or adversely. Accordingly, we should monitor the safety of probiotics as our research efforts move forward.

Our analysis has several limitations. First, as already mentioned, there was heterogeneity in the inclusion criteria, the populations studied, the probiotic agents used, doses, time points when therapy was initiated, durations of therapy, the routes of administration, and the diagnostic criteria used for establishing NP or VAP. These factors were not comparable in most of the trials and might have affected the clinical outcomes. These differences may explain the statistical heterogeneity in some of the secondary outcomes investigated. Second, even though we were able to pool results across all trials, the number of patients included in this meta-analysis may not be sufficient to exclude significant clinical benefit. Third, most trials were done in single centers and may have had inherent bias related to local practice habits and the populations served. Although we extensively searched for relevant studies using multiple databases and multiple search items, and no language restriction was placed on the search, a funnel plot suggested the possibility of publication bias. Finally, the quality of the included studies was not consistent. Some RCTs included in our analysis had major methodological flaws [24,28]. The quality of trials can affect the direction and magnitude of treatment effects when performing a meta-analysis.

Although the results have been encouraging, there is insufficient evidence to suggest to clinicians that administration of probiotics is associated with significant clinical benefit in critically ill patients. In addition, there is a lack of head-to-head comparative trials with different probiotics to investigate and clarify which probiotic bacterial strains were of most benefit to critically ill patients. Data regarding the superiority of different doses of probiotics and routes of administration are also lacking. The questions that remain to be evaluated in large-scale, randomized controlled trials of probiotics use in NP include the optimal type of probiotic preparation, administration route, dose intensity, timing and duration of administration, safety, patient eligibility, and contraindications.

## Conclusions

The use of probiotics was associated with statistically significant reduction in the incidence of NP in critically ill patients. However, there is no evidence to support or refute claims of beneficial effects on clinically important outcomes. Large, well-designed, randomized, multi-center trials are needed to confirm the effects of probiotics in diverse populations of critically ill patients.

## Key messages

• Numerous studies have examined the utility of probiotic therapy to prevent NP in critically ill patients, but studies on the use of probiotics have yielded mixed results.

• Probiotics administration was associated with statistically significant reduction in the incidence of NP in critically ill patients.

• More randomized control trials are needed to definitively determine the effect of probiotics in critically ill patients.

## Abbreviations

APACHE: Acute Physiology and Chronic Health Evaluation; CI: confidence interval; DB: double-blind; MC: multicenter; MD: mean difference; MV: mechanical ventilation; NA: not available; NP: nosocomial pneumonia; OR: odds ratio; RCTs: randomized control trials; SAPS: Simplified Acute Physiology Score; SC: single-center; T: temperature; VAP: ventilator-associated pneumonia.

## Competing interests

The authors declare that they have no competing interests.

## Authors' contributions

KXL and YGZ carried out the primary study search, extracted data, performed statistical analysis, and drafted and revised the manuscript. JZ drafted and revised the manuscript. LLT carried out statistical analysis and revised the manuscript. JWL revised the manuscript and modified the written English. XDW carried out statistical analysis and helped draft the manuscript. JMQ conceived the idea, participated in its design, and drafted and revised the manuscript. All authors read and approved the final manuscript.

## Supplementary Material

Additional file 1 **Forest plot showing the effect of probiotics on the occurrence of ventilator-assisted pneumonia in critical ill patients receiving mechanical ventilation**. Click here for file

Additional file 2 **Forest plot showing the effect of probiotics on the occurrence of nocosomial pneumonia in l critically ill surgical patients**. Click here for file
